# Colorimetric determination of cysteine and copper based on the peroxidase-like activity of Prussian blue nanocubes

**DOI:** 10.1039/d1ra06838e

**Published:** 2021-11-18

**Authors:** S. Kavitha, S. Mary Jelastin Kala, A. Anand Babu Christus, A. Ravikumar

**Affiliations:** Research and Department of Chemistry, St. Xavier's College (Affiliated to Manonmaniam Sundaranar University, Abishekapatti, Tirunelveli-627012, Tamil Nadu, India) Tirunelveli-627002 Tamil Nadu India kavithasundar2010@gmail.com +91 9486558124; Research and Department of Chemistry, St. Xavier's College (Affiliated to Manonmaniam Sundaranar University, Abishekapatti, Tirunelveli-627012, Tamil Nadu, India) Tirunelveli-627002 Tamil Nadu India; Department Chemistry, SRM Institute of Science and Technology, Ramapuram Campus Ramapuram-600089 Chennai Tamil Nadu India; General Practice Center, The Seventh Affiliated Hospital, Southern Medical University Foshan 528244 P. R. China; Institute of Environment and Health, South China Hospital, Health Science Center, Shenzhen University Shenzhen 518116 P. R. China

## Abstract

Prussian blue nanocubes were synthesized *via* a hydrothermal method. Significantly, the redox couple Ni^3+^/Ni^2+^ provided rich oxidation and reduction reactions, which enhance catalytic activity. Furthermore, PBNCs mimic peroxidase activity which could oxidise colourless tetramethyl benzidine (TMB) to a blue colour (TMB^+^) in the presence of H_2_O_2_. Thus, it can be used as a colorimetric sensing platform for detecting cysteine and Cu^2+^. The addition of cysteine to a TMB + PBNCs sensing system decreases the intensity of the blue colour in the solution with a decrease in the absorption peak at 652 nm in the UV visible spectrum. Subsequently, the addition of Cu^2+^ into the TMB + PBNCs + Cys sensing system increases the intensity of the blue colour due to complex formation of Cu and cysteine. Therefore, the change in intensity of the blue colour of TMB is directly proportional to the concentration of Cys and Cu^2+^. As a result, this sensing system is highly sensitive and selective with an effective low detection limit of 0.002 mM for cysteine and 0.0181 mM for Cu^2+^. Furthermore, this method was applied to the detection of cysteine and copper in spiked real samples and gave satisfactory results.

## Abstract 2

Prussian blue nanocubes were synthesized *via* a hydrothermal method.

## Graphical abstract

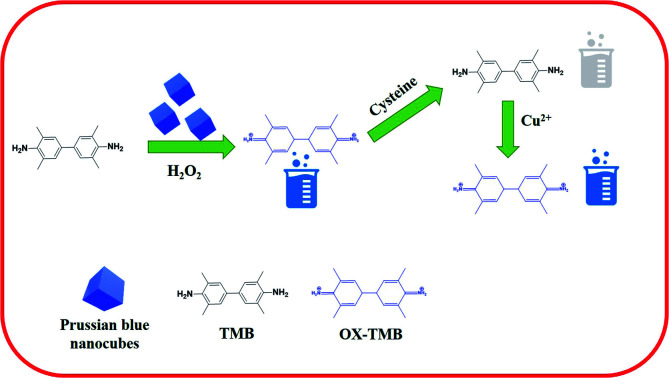

## 1. Introduction

The detection of heavy metal ions in natural aquatic systems is challenging work for contemporary researchers. Even trace levels of heavy metal are highly toxic in nature and can spread through food chains causing severe health hazards to humans and other species on the earth.^1,2^ Copper is one of the most vital nutrient metals responsible for performing many cellular reactions in living beings, but when it exceeds its permissible level, it produces toxicity to the immune system and also leads to DNA rupture, carcinogenicity and neurological disorder to the health system, Alzheimer's disease, Parkinson's disease, Wilson's disease, *etc.*^3–13^

Recently, different conventional methods have been employed in the detection of copper(ii) ions based on atomic absorption spectrometry (AAS)^14,15^ inductively coupled plasma atomic emission spectrometry (ICP-AES),^16,17^ inductively coupled plasma mass spectrometry,^18^ laser ablation inductively coupled plasma mass spectrometry (LAICP-MS),^19^ X-ray fluorescence spectroscopy (XRF),^20^ anodic stripping voltammetry (ASV),^21^ photo-brightened luminescence (PBL),^22^ and ion chromatography-ultraviolet and visible spectrophotometry (IC-UV).^23^ Even though these techniques are highly sensitive in detection, there are some drawbacks to these techniques, such as the need for sophisticated instrumentation, complicated procedures and the requirement for trained technicians, which limit the operation of these techniques. So it is timely to find an alternative method to detect the copper(ii) ions in aqueous solution, which should be convenient, simple, and with portable facilities for infield samples. The colorimetric method is more suitable for the detection of Cu^2+^ ions, due to its uncomplicated, cheap and quick detection process as the colour change can be observed by the naked eye.^24–29^

Currently, enzyme-mimicking catalytic active reactions in colorimetric analysis have received a great deal of attention in the detection of heavy metal ions and other biomolecules. Peroxidase-mimicking nanoparticles are highly efficient and superior in properties over natural enzymes in their stability and sturdiness under tough reaction conditions. Also, they are cheap and easy to prepare. In the emerging development of nanotechnology, many novel nanomaterials have been synthesized with intrinsic mimic-like activity, behaving as mimicking enzymes in the place of established enzymes. A variety of nanomaterials, such as metals (Cu^2+^),^30^ bimetals (Au@Pt, Au@Pd),^31,32^ metal oxides (CuO, CeO_2_),^33,34^ metal sulfides (MoS_2_),^35^ metal organic frameworks MOFs,^36^ carbon-based nanomaterials,^37–40^ Au nanoclusters^41^ and TiO_2_ nanotube arrays,^42^ have been proved to possess peroxidase-mimicking activity. Among these, Prussian blue (PB) based materials have been extensively applied in different fields, such as electrochemical sensors,^43–45^ fiber optic gas sensors,^46^ the oxygen reduction reaction,^47^ and lateral flow assays.^48^ In recent years it was found that PB nanocubes as a prototype for transition metal hexacyanoferrates have been reported to have highly intrinsic peroxidase-like activity due to the highly active formation intermediates of metals and oxygen, which is similar to a heme structure. However, few colorimetric sensors based on PBNCs have been reported with good peroxidase activity and high sensitivity.

In this work, we effectively utilize the intrinsic catalytic property of PBNCs to detect cysteine and Cu^2+^ in solution. The synthesized PBNCs catalyze the oxidation of tetramethyl benzidine (TMB) in the presence of H_2_O_2_ to form TMB^+^ radical ions in solution, which turn the colour of the solution blue. The incorporation of a cysteine decolourised solution due to its cation restoration property helps to detect the concentration of cysteine in the solution. Upon addition of Cu^2+^ to the same solution, it regains the blue colour due to the affinity between a copper ion and the thiol group of cysteine. Based on this strategy, a novel colorimetric sensing system was developed for the highly selective and sensitive detection of cysteine and Cu^2+^. To the best of our knowledge, the effective peroxidase mimicking activity of a PBNC based colorimetric sensing system has been used for the first time for the detection of cysteine and copper and evaluated with spiked real water samples in aqueous solution.

## 2. Experimental section

### 2.1. Materials

All the chemicals and solvents used in this experiment were purchased commercially. Sodium citrate, nickel nitrate, potassium hexacyanocobaltate(iii), ethanol, 3,3′,5,5′-tetramethylbenzidine (TMB), hydrogen peroxide (H_2_O_2_, 30%) lead nitrate, cobalt nitrate, nickel nitrate, zinc nitrate, silver nitrate, ferric nitrate, copper chloride, calcium chloride, ferrous chloride, manganese chloride, magnesium chloride, cadmium chloride, and barium chloride, and cysteine (Cys), glutathione (GSH), histidine (His), glucose (Glu), methionine (Met), phenylalanine (Phe), valine (Val), proline (Pro), arginine (Arg), thyamine (Thy), homocysteine (Hcy) and uric acid (Ura) were obtained from Sigma Aldrich and Alfa Aesar in India.

### 2.2. Preparation of Prussian blue nanocubes

Firstly, Ni (NO_3_)_2_ (12.9 mg) and sodium citrate (0.3 g) were dispersed using 40 mL of deionized water and ethanol (v/v = 1 : 3), and stirring was continued to form a transparent solution. After that, 10 mL of K_3_[Co (CN)_6_] (13.2 mg) solution was added dropwise into the above solution and stirred for 30 min and then the reaction continued up to 12 h. Finally, the obtained precipitate of Prussian blue nanocubes was washed several times using ethanol and water and dried at room temperature for 6 h.

### 2.3. Colorimetric sensor for Cys and Cu^2+^ based on peroxidase activity

A colorimetric detection system for Cys and Cu^2+^ was constructed based on the peroxidase activity of PBNCs as enzyme mimics. In a typical procedure, 7 μL of PBNCs (7 μL mL^−1^), 50 μL of H_2_O_2_ and 50 μL of TMB solution were mixed with acetate buffer (938 μL) solution. After that, the mixed solution was kept for 3 min at room temperature, 5 μL of Cys of different concentrations were added and incubated for 15 min to form TMB + PBNCs + Cys. The absorption intensity was measured using a UV-Visible spectrophotometer at a wavelength of 652 nm. After that, for the fluorescence detection of Cu^2+^, PBNCs (7 μL), H_2_O_2_ (50 μL), TMB (50 μL) and Cys (50 μL) were mixed and diluted with buffer to a final volume of 1 mL and incubated for 15 min. Next, different concentrations of Cu^2+^ were added to the solution. The absorption intensity was measured at a wavelength of 652 nm.

## 3. Results and discussion

### 3.1. Characterization of PBNCs

**Fig. 1:**
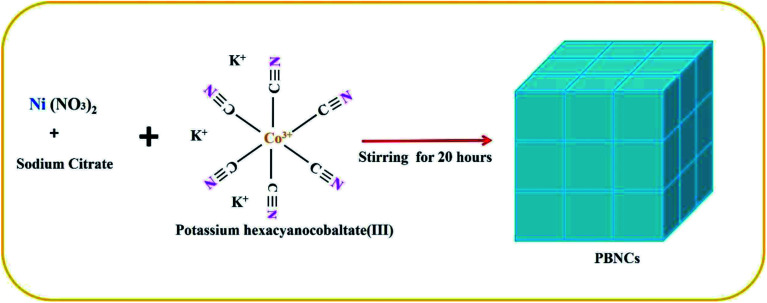
Graphical representation of Prussian blue nanocube preparation.

**Fig. 2:**
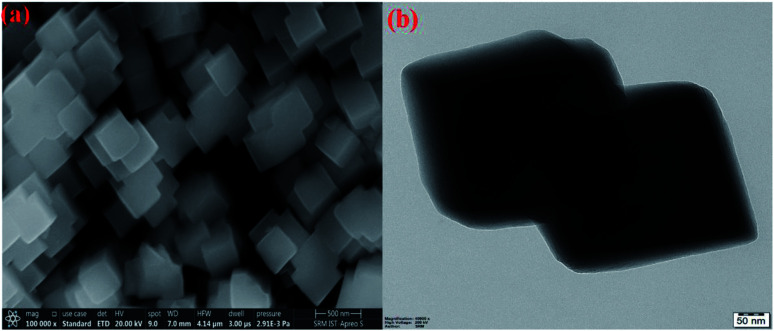
HR-SEM and HR-TEM (a and b) images of PBNCs.

The preparation of PBNCs, as shown in Fig. 1, and the morphology of the prepared materials were studied by scanning electron microscopy (SEM) and transition electron microscopy (TEM). Fig. 2a and b show the regular cubic shape with a smooth surface and high uniformity of the prepared PBNCs with an average particle size of around 320 nm, which consist of a large number of cubic formations. In general, the decrease in particle size leads to more active sites, which results in an enhancement in catalytic activity.

**Fig. 3:**
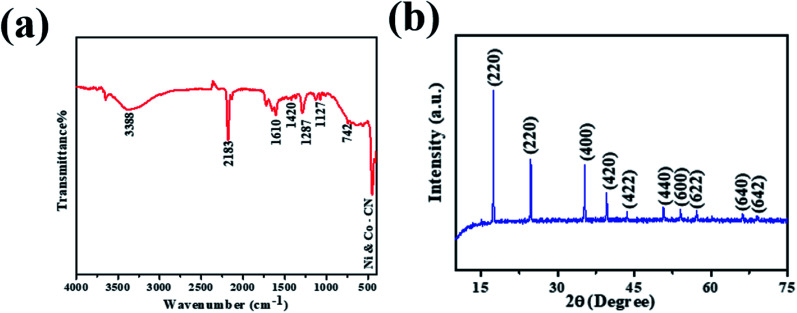
FT-IR spectra and XRD images of PBNCs.

The crystalline structure and phase composition of PBNCs were explained through X-ray diffraction (XRD). As shown in Fig. 3a, the diffraction pattern of PBNCs is well matched with a previous report.^49^ The diffraction peaks centered at approximately 2*θ* = 17.2, 24.5, 35.4, 39.9, 43.5, 50.7, 54.2, 66.4 and 69.2 were indexed to the (200), (220), (400), (420), (422), (440), (600), (620), (640) and (642) crystal planes, as evidence for the successful synthesis of PBNCs. Furthermore, the FT-IR spectrum was employed to analyze the functional groups of PBNCs and the obtained results are displayed in Fig. 3b. The characteristic peak at 2181 cm^−1^ was accredited to the CN stretching vibration of the Ni^2+^–CN–CO^3+^ complex. The absorption peaks at 562 and 453 cm^−1^ are attributed to Co–CN and Ni–CN. The absorption peak corresponding to the NH/OH bending mode at 3388 cm^−1^ was also found. The above results further indicate the formation of PBNCs.

### 3.2. Peroxidase-like activity of PBNCs

**Fig. 4:**
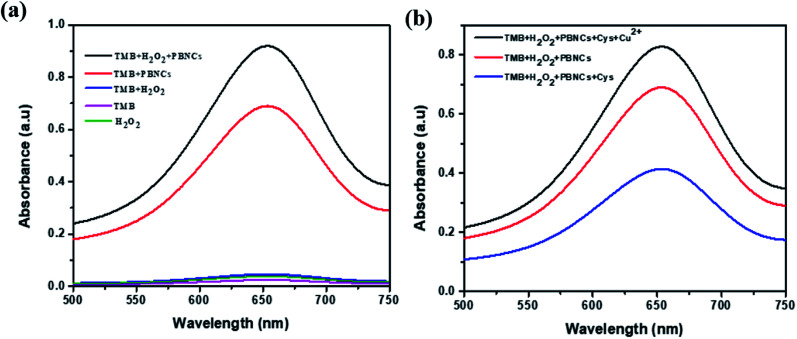
(a) UV-vis absorption spectra of PBNCs/TMB/H_2_O_2_, PBNCs/TMB, TMB/H_2_O_2_, TMB, and H_2_O_2_. (b) Typical absorption spectra of different systems: TMB + H_2_O_2_ + PBNCs, TMB + H_2_O_2_ + PBNCs + Cys, TMB + H_2_O_2_ + PBNCs + Cys + Cu^2+^.

The peroxide-mimicking activity of the synthesized PBNCs was investigated with UV visible spectra. As shown in Fig. 4a, the UV spectra of the test samples containing pure H_2_O_2_, pure TMB and a mixture of both (H_2_O_2_ + TMB) show zero absorption intensity at 652 nm. PBNCs mixed with pure TMB and mixed with both (H_2_O_2_ + TMB) show a larger absorption peak compared with pure TMB, and PBNC cubes produce a maximum absorption peak at 652 nm for the mixture containing H_2_O_2_ and TMB. The results strongly proved that the synthesized PBNC cubes possess good peroxidase-mimicking activity with the H_2_O_2_ + TMB system.

**Scheme 1:**
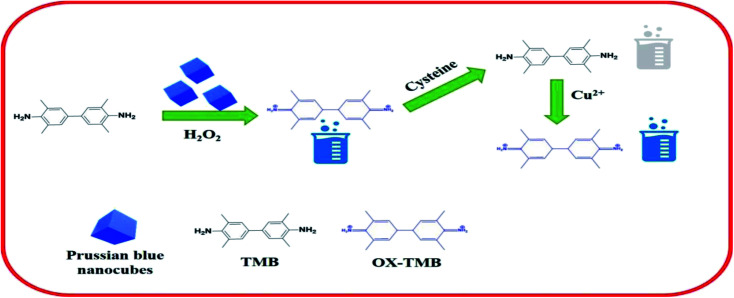
Schematic representation of the sensing mechanism for the detection of cysteine and copper based on PBNCs.

Scheme 1 represents the schematic sensing mechanism involved in the detection of cysteine and Cu^2+^ in aqueous solution based on the peroxidase-mimicking activity exhibited by the PBNCs. The synthesized PBNCs activate the oxidation of TMB molecules in the presence of H_2_O_2_ in the solution and convert them to their radical ions (TMB^+^) which turn the colour of the solution deep blue. When the biomolecule cysteine is incorporated into the solution, it rapidly decolourises the solution due to its anti-radical property, which hinders the formation of TMB radical ions. A stable nanozyme–cysteine complex is formed as soon as the PBNCs nanozyme is exposed to l-cysteine. The formation of this stable complex will significantly deplete the population of free l-cysteine (inhibitor) in the reaction. The inhibitors bind to the natural enzyme with an apparent affinity close to the concentration of the active sites of the enzyme. Addition of Cu^2+^ ions into the solution gets back its blue colour. This is due to the strong affinity of a Cu^2+^ ion to the thiol group of cysteine that suppresses the anti-radical effect of the cysteine and enhances the oxidation of the TMB molecules in the solution.^50,51^

**Fig. 5:**
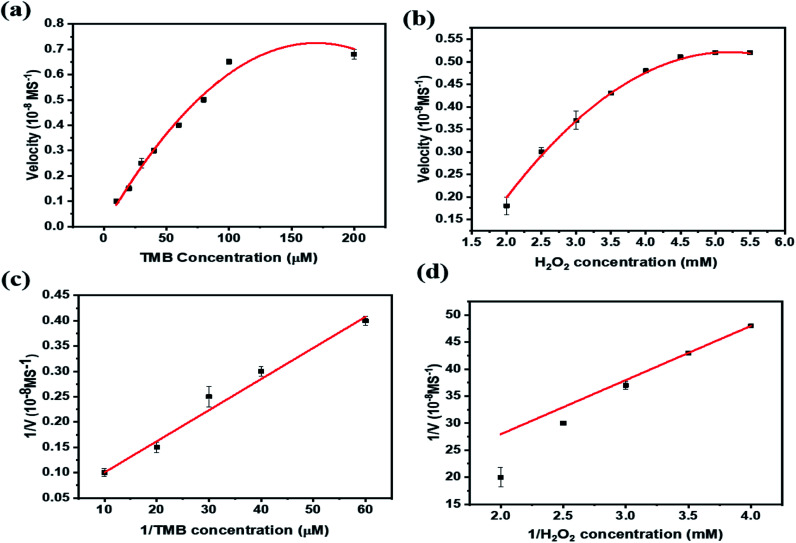
Steady-state kinetic analysis of PBNCs in changing concentrations of TMB and H_2_O_2_: (a and b), corresponding double reciprocal plots for TMB and H_2_O_2_ (c and d).

As shown in Fig. 4b, PBNCs can catalyze the oxidation of TMB in the presence of H_2_O_2_ to generate the blue colour with good absorption intensity (TMB + PBNCs + H_2_O_2_). With injection of cysteine into the above system, the absorption intensity decreases and slows down the catalytic activity due to cysteine molecules capturing the hydroxyl radicals (TMB + PBNCs + H_2_O_2_ + Cys). However, the catalytic activity of PBNCs quickly increases with the addition of Cu^2+^ ions to the reaction system because the thiol moiety of the cysteine molecule interacts with Cu^2+^ to form a thiol–copper complex. Based on the results, it is confirmed that the synthesized PBNCs possess very good peroxidase-like activity which can be used as a colorimetric sensor for the detection of cysteine and copper in solution. To better investigate the peroxidase-like catalytic activities of PBNCs, steady-state kinetic tests were conducted with different concentrations of TMB in the absence and presence of H_2_O_2_. The Michaelis–Menten curves (Fig. 5a and b) and Lineweaver plots (Fig. 5c and d) were built on reaction velocities and TMB and H_2_O_2_ concentrations. It could be clearly seen that all the fitting curves showed good correlation and the *K*_m_ values were calculated. The *K*_m_ values were measured to be 0.0312 and 0.025 mM in the presence and absence of H_2_O_2_, respectively. The results were lower than the results obtained in the presence of horseradish peroxidase.^52^ Based on these results, the prepared PBNCs with good peroxidase-like activity possessed a high affinity towards H_2_O_2_ and TMB.

### 3.3. Optimization

**Fig. 6:**
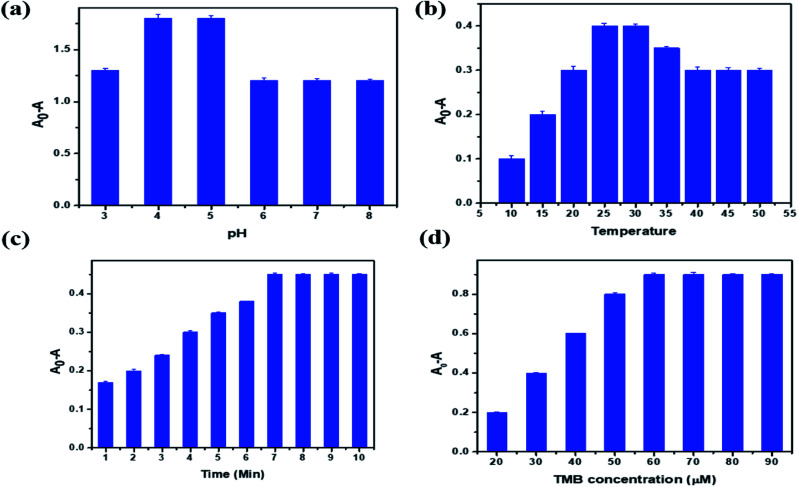
Effect of (a) pH, (b) temperature, (c) time, (d) TMB concentration.

Optimization of reaction parameters is necessary to study the efficiency of the proposed sensor in the detection of a target analyte. In the optimization of PBNC, solution pH, sample temperature, reaction time and concentration of TMB were chosen. Fig. 6a represents a graphical plot of relative activity with respect to the pH of the sample. From the figure it is clear that the reaction takes place in an acidic medium: as the pH of the solution increases from 3 to 5, the relative activity also increases. After pH 5 there is no change in activity due to a decrease in substrate oxidation in the TMB molecule. Hence, pH 4.5 was selected as the optimum pH for the sensing system. Activity with respect to temperature is plotted in Fig. 6b. This shows that as the temperature of the reaction mixture increases from 10 to 30 °C, the relative activity also increases and then decreases. This is because at higher temperature H_2_O_2_ is unstable, which affects the oxidation of TMB molecules. So the optimum temperature for the reaction to be maintained was 28.2 °C.

In the same manner, the incubation time was studied. The absorbance gradually increases up to 7 min, as shown in Fig. 6c; then it remains the same, because when the oxidation of TMB is completed there is no more TMB available in the sample solution. Fig. 6d shows the optimization graph for the TMB concentration. The absorption increases as the concentration of TMB increases from 20 to 60 μM. Then it remains constant in the higher concentration solution because the available concentration of PBNC was completely utilized for the oxidation of TMB molecules up to 60 μM, and beyond this limit there are no more catalytic agents to oxidize the available TMB molecules in the test solutions. Based on all these observations in the optimization study, the optimal values suggested for pH, temperature, time and concentration of TMB are 4.5, 28.2 °C, 7 min and 60 μM, respectively.

### 3.4. Sensitivity and selectivity for cysteine

**Fig. 7:**
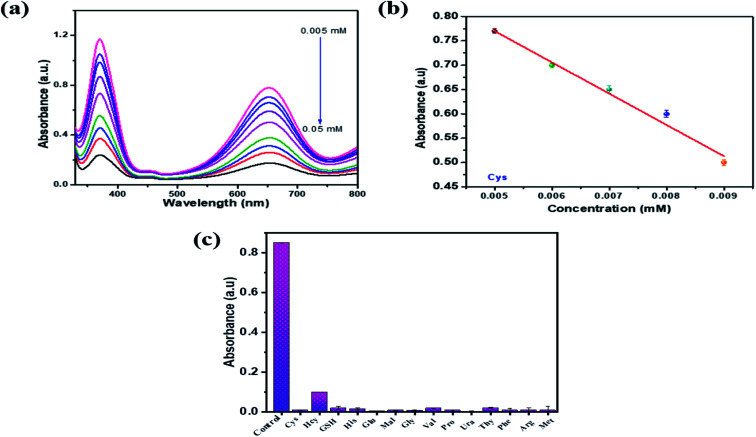
Cysteine detection using PBNCs as a peroxidase mimic. (a) Cysteine concentration dependent change in UV-absorption intensity. (b) The calibration curve of cysteine detection obtained from UV-absorption spectra. (c) Selectivity study of PBNCs for the detection of cysteine.

To examine the sensitive detection of cysteine by PBNC, different concentrations of Cys (0.005 –0.5 mM) were added to test solutions containing TMB, H_2_O_2_, sodium acetate buffer and PBNC at optimum levels and incubated for 7 min. After the incubation period, UV absorption spectra were taken, as shown in Fig. 7a. It was observed that, as the concentration of Cys increases in the test solution, the absorption decreases due to hindrance in the oxidation of TMB molecules by Cys. As a result, the deep blue colouration of the test solutions fades in the higher concentration of Cys. Fig. 7b represents a graphical plot of absorption *vs.* Cys concentration. A good decrease in the linear plot was observed between the concentration range (0.05–0.009 mM) of Cys and their linear correlation coefficient was about 0.993. The limit of detection (LOD = 3*σ*/*S*, *σ*: standard deviation, *S*: standard curve slope) values for cysteine were calculated to be 0.002 mM. Selectivity for Cys and other biomolecules (Fig. 7) were investigated with PBNC, as shown in Fig. 7c. Different test samples were prepared with optimal levels of TMB, H_2_O_2_ and PBNC. To this test solution Cys and other biomolecules (His, Hcy, GSH, Ura, Glu, Phe, Met, Thy, Gly, Pro, Arg, Mal, Val) were added and all the solutions were kept for incubation. A test solution containing only TMB, H_2_O_2_ and PBNC was considered as a blank solution. After the incubation period UV absorption spectra were taken for the blank and other test samples. It was observed that only the blank sample shows the maximum absorption peak whereas other biomolecules show very low absorption and Cys showed nil absorption in the UV spectra.

### 3.5. Sensitivity and selectivity for copper

**Fig. 8:**
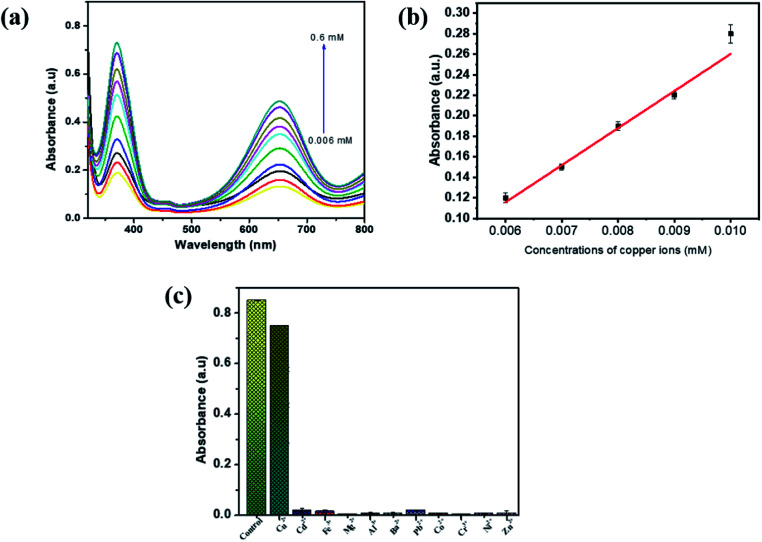
Cu^2+^ detection using PBNCs as peroxidase mimic. (a) Cysteine concentration dependent change in UV-absorption intensity. (b) Calibration curve of Cu^2+^ detection obtained from UV-absorption spectra. (c) Selectivity study of PBNCs for the detection of Cu^2+^.

The synthesized PBNC was subjected to a sensitivity and selectivity study for Cu^2+^ in the solution. For the investigation of selectivity, sample solutions were prepared with optimum levels of TMB, H_2_O_2_, sodium acetate buffer and PBNC. To this solution 0.5 mM of Cys was added to all test samples and incubated for 7 min. After the incubation period, Cu^2+^ were added to the prepared sample solution in the range from 0.006 mM to 0.6 mM and incubated for another 7 min. Then UV absorption spectra were taken for all the test samples at 652 nm, as shown in Fig. 8a. The absorption spectra show there was an increase in UV absorption as the concentration of Cu^2+^ ions increased in the sample solutions. This is because when Cu^2+^ ions become bound with Cys it reduces the anti-radical property of the Cys and enhances the oxidation of TMB molecules, and as a result the absorption will increase in the sensing system. Fig. 8b shows the graphical plot between absorption and concentration of Cu^2+^. Good linearity was observed between 0.006 and 0.009 mM of Cu^2+^ and their linear correlation coefficient was about 0.975. The limit of detection (LOD = 3*σ*/*S*, *σ*: standard deviation, *S*: standard curve slope) value for cysteine was calculated to be 0.0181 mM. For a selectivity study of PBNC, 2 mL of sample solution were prepared containing an optimum level of TMB, H_2_O_2_, sodium acetate buffer, PBNC and Cys, and to this various metal ions were added at 0.5 mM, and one blank sample was prepared without the addition of Cys and metal ions. All the samples were incubated for 7 min. and absorption spectra were taken, and the result suggested that only Cu^2+^ ions show maximum absorption compared with other metal ions, such as Cd^2+^, Fe^3+^, Mg^2+^, Al^3+^, Ba^2+^, Pb^2+^, Co^2+^, Cr^2+^, Ni^2+^, Zn^2+^, suggesting that the proposed colorimetric sensor possesses good selectivity in the determination of Cu^2+^ in aqueous solution (Fig. 8c).

### 3.6. Application of real sample analysis in Cu^2+^

In order to investigate the practical application of the proposed sensing system and to detect cysteine and Cu^2+^ in Tamirabarani river water and tap water, real water samples were collected from river water near our college campus and tap water in our lab. There was no detectable cysteine or Cu^2+^ in the real water samples used for colorimetric sensing, so different concentrations of the above were prepared and spiked in collected real water samples. It was found that the result obtained by the PBNC based colorimetric sensing system was verified to determine the precision of this method. The observed results are summarized in Table 1. These results indicated that the proposed colorimetric sensor can be used for real water sample analysis for cysteine and Cu^2+^.

**Table 1:** Analysis of real water samples in cysteine and Cu^2+^ sensing with a PBNC based colorimetric sensor

Sample	Spiked (nM)	Detected ± SD		Recovery (%)	
		Cysteine	Cu^2+^	Cysteine	Cu^2+^
River water	0.02	0.0196 ± 0.0001	0.0190 ± 0.0005	98	95.5
	0.04	0.0386 ± 0.0001	0.041 ± 0.0006	96.5	102
	0.06	0.0598 ± 0.0001	0.0599 ± 0.0002	99.6	99.8
Tap water	0.02	0.0195 ± 0.0001	0.021 ± 0.000076	97.5	100.5
	0.04	0.0398 ± 0.00015	0.0369 ± 0.0001	99.5	99.5
	0.06	0.0595 ± 0.00018	0.062 ± 0.0002	99.1	103

## 4. Conclusion

In this work, uniformly structured PBNCs with excellent peroxidase activity were synthesized by a hydrothermal method. PBNCs exhibit high peroxidase activity and could oxidize the colourless TMB to blue TMB. The proposed colorimetric sensing system of TMB + PBNCs could achieve excellent sensitivity and selective detection of cysteine with a detection limit as low as 0.002 mM in the linear range 0.005–0.009 mM. Furthermore, the sensing system of TMB + PBNCs + Cys could realize the detection of Cu^2+^ (0.006–0.009) with a low detection limit of 0.007 mM. Interestingly, this colorimetric sensing system shows a low detection limit and good linear range, and exhibits good accuracy and satisfactory recovery results in real samples. Therefore, the prepared PBNCs act as a good platform for the detection of cysteine and copper ions in real-time environmental and biological applications.

## Conflicts of interest

There is no conflicts of interest.

## Supplementary Material
